# Clinicopathological report on epidermoid cysts of the brain: A case series and literature review from an African perspective

**DOI:** 10.1016/j.ijscr.2023.107969

**Published:** 2023-03-10

**Authors:** Moshawa Calvin Khaba, Nomthandazo Amanda Dube

**Affiliations:** a Department of Anatomical Pathology, Dr George Mukhari Academic Laboratory, National Health Laboratory Service, Sefako Makgatho Health Sciences University, Ga-Rankuwa, South Africa; b Department of Neurosurgery, Dr George Mukhari Academic Hospital, Sefako Makgatho Health Sciences University, Ga-Rankuwa, South Africa

**Keywords:** Brain, Epidermoid, Keratinous cyst, Africa

## Abstract

**Introduction:**

Epidermoid cysts are rare benign lesions of the central nervous system which accounts for approximately 1–2 % of all intracranial tumours. They are commonly located in the parasellar region, cerebellopontine angle; however, brain parenchyma origin is rare. We report clinicopathological features of these rare lesions.

**Method and material:**

This is a retrospective study of epidermoid cyst of the brain diagnosed between 01 January 2014 and 31 December 2020.

**Results:**

The four patients had mean age of 30,8 years (range: 3–63), one male and 3 females. All four patients presented with headache and one associated with seizures. Radiological images showed two posterior fossa; each occipital and temporal locations. All tumours were successfully removed and histopathological assessment confirmed epidermoid cysts. All patients showed clinical improvement and were discharged home.

**Conclusion:**

Epidermoid cysts of the brain are rare and still remain a preoperative clinico-radiological conundrum as they may be indistinguishable from other intracranial tumours. Therefore, collaboration with histopathologists is advised in the management of these cases.

## Highlights

• Epidermoid cysts of the brain are usually rare.
• They are a clinical and radiological challenge, especially in experience hands.
• Accurate management is associated with good clinical outcome.
• Epidermoid cysts of the brain in Africa are underreported.

## 1 Introduction

Epidermoid cysts (EC) are rare benign lesions of the brain which are estimated below 2 % of all brain tumours [1], [2], [3]. These lesions are usually considered congenital as they are formed around the 3rd and 5th weeks of intrauterine life due to abnormal trapping of ectodermal cells. This eventually develops in to the epidermis within the nervous tissue during neural tube closure.

Around this period of embryogenesis, the otic and optic vesicles are also being formed. Therefore, the inclusion of ectodermal cells within these structures has been attributed to the cerebellopontine angle and parasellar region being the common site of epidermoid cyst of the brain [2], [3], [4]. Other sites of EC include parapontine region, middle cranial fossa, parapituitary region, diploe, and spinal canal while pure intracerebral epidermoids are rare [1], [4], [5].

Accurate preoperative radiological diagnosis remains problematic because of the close similarity to more common intracranial cystic tumours. Moreover, the accurate preoperative diagnosis of intraparenchymal ECs is more important because they may result in chemical meningitis during or after operation. Based on the literature search, only 7 cases have been described from the African continent exclusively from three countries [1], [6], [7], [8], [9], [10]. This case series has been reported in line with PROCESS guideline [11].

## 2 Methods and material

This is a retrospective study of cases diagnosed with epidermoid cyst of the brain for a period of 6 years (01 January 2014–31 December 2020) in our centre. Departmental and hospital records of these patients were accessed, where available, to record clinical details age (gender, age, race, clinical presentation and clinical outcomes). Radiological investigations where available and haematoxylin and eosin (H&E) stained sections were retrieved and reassessed.

## 3 Results

See Table 1 for clinicopathological findings.

**Table 1:** Clinicopathological features.

Case	Age (years)	Gender	Clinical presentation	Imaging	Histopathological diagnosis	Outcome
1	63	Male	Headache and seizures with associated hydrocephalus	Cerebellopontine angle	Epidermoid cyst	Asymptomatic on follow-up visits
2	30	Female	Headache and inability to walk	4th ventricular tumour	Epidermoid cyst	Asymptomatic on follow-up visits
3	27	Female	Headache and seizures.	Cerebral tumour in the temporal lobe	Epidermoid cyst	Asymptomatic on follow-up visits
4	3	Female	Headache and seizures with loss of weight	Cerebral tumour in the occipital lobe	Epidermoid cyst	Asymptomatic on follow-up visits

**Fig. 1:**
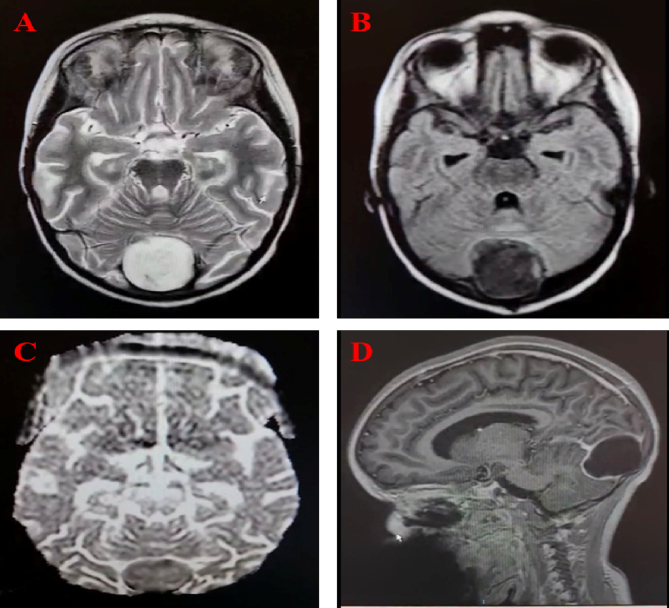
A-D: MRI of the brain (case 4) show a well circumscribed cystic lesion in the posterior fossa which causes splaying of the superior sagittal sinus and transverse sinuses bilaterally.

**Fig. 2:**
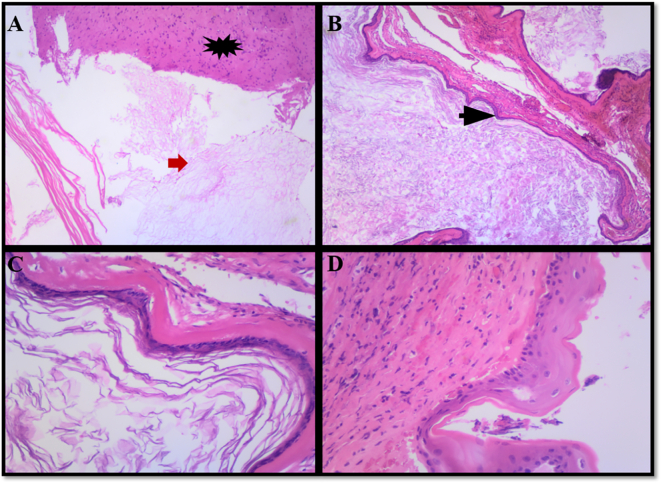
A–D show brain cyst. Brain (black star); Keratin (red arrow); stratified squamous epithelium (black arrow). (For interpretation of the references to colour in this figure legend, the reader is referred to the web version of this article.)

The study consisted of four patients composed of one male and 3 females with a mean age of 30,8 years (range: 3–63). All four patients presented with headache. From this, only one patient has associated seizures. Imaging reports were accessed on 3 cases and was not possible to review the images (case1–3). Based on brain imaging, 2 patients had posterior fossa, 1 had occipital and 1 had temporal tumours (Fig. 1). Two patients with posterior fossa tumours had associated hydrocephalus. All tumours were successfully removed and histopathological assessment confirmed epidermoid cysts (Fig. 2). All patients showed clinical improvement and were discharged from the neurosurgical unit to their respective homes.

## 4 Discussion

Although they are considered congenital, they do not manifest until the 2nd to the 6th decades of life due to their slow growth. Their increase in size is due to accumulation of keratin and cholesterol arising from the desquamation of epithelial cells. Furthermore, their late presentation may be attributed to the absence of mass effect due to these tumours having propensity to grow along available cisternal spaces [11], [12]. This is evident in this study as three patients presented from the second decade of life to the 6th decade. However, one patient that presented early at 3 years. The size the tumour at 3 years gives an impression that it had accelerated growth. This is very uncommon and was highlighted by only 1 paediatric case been reported from Africa to date (Table 2) [1], [6], [7], [8], [9], [10]. Most of the reported cases from Africa are single case reports with Balogun et al., 2018 having reported 2 cases. To date, this study has the largest cohort from the African continent.

**Table 2:** Reported cases in Africa.

Authors	Year	Number of cases	Location	Age	Outcome	Country
Aggouri et al., 2009	2010	1	Fourth ventricle	Adult		Morocco
Hassani et al., 2014	2014	1	Pineal gland	Adult		Morocco
El Mghari et al., 2017	2017	1	Sella turcica	Adult		Morocco
Balogun et al., 2018	2018	2	Cerebella vermian	Adult		Nigeria
Africha et al., 2019	2019	1	Extradural	Adult		Morocco
Labuschagne et al., 2020	2020	1	Brain stem	Paediatric		South Africa

Epidermoid cysts are extra-axial lesions, commonly found in the posterior fossa, with the cerebellopontine angle cistern being the most common location [14]. All four cases in this study had different locations including with only one arising from the cerebellopontine angle. While the cerebellopontine angle might not be the common location in this study, the limitation of this study and exististing African literature is the small sample size and one cannot comment on the statistical significance.

Due to their slow growth, clinical symptoms of BEC are usually due to insidious mass effect with or without cranial nerves involvement which happen after many years of life. Mass effect is responsible for most symptoms as the cyst contents accumulation may cause direct compression of the brain structures [13], [15].

The common symptom is headache followed by seizures, cranial nerve abnormalities, cerebellar symptoms and raised intracranial pressure in no particular order. Hydrocephalus has also been described in other reports [12], [13], [15]. Headache was present in all the cases in this study (Table 1).

Radiological imaging is instrumental in establishing the diagnosis of EC with computed tomography (CT) and magnetic resonance imaging (MRI) the preferred choice. However, MRI is the imaging modality of choice in these lesions [13]. The CT scan show a well-defined lobulated mass which is hypodense while on MRI is hypointense on T1-weighted and hyperintense on T2-weighted and DWI [13], [14]. The retrospective nature of this study made it difficult to access all the radiological data needed, therefore, precluding accurate reporting and/or interpretation of the radiological images and/or findings (Fig. 1).

Histopathological assessment is an essential tool in establishing the exact origin of the brain cysts. Grossly, EC are well-circumscribed tumours with a shiny nodular surface with keratinous contents on cut surface and occasional calcifications [14] 11. Microscopically, they are lined by stratified squamous epithelium with granular cell layer and luminal keratin [14] 9. In case of cyst rupture, foreign body giant cell reaction, inflammation and/or abscess formation may be seen 9. The cyst lining and wall compositions are important in the differentiating them from the other cysts.

The following cysts should be considered in the differential diagnosis of brain EC: colloid cysts, arachnoidal cysts, dermoid cysts, neurocystocercosis, neuroenteric cyst. Cystic tumours of the brain and hamartomatous lipomas should also be considered.

These tumours can be differentiated by imagining and confirmed by histopathology [12].

Radiology and histopathology are important in reaching the correct diagnosis which will allow the surgeons to plan for adequate and/or definitive management.

The primary goal of treatment is to completely remove the cyst and its contents. EC are very troublesome to completely remove as they growth into different spaces and cisterns. Furthermore, they tend to engulf cranial nerves and vessels making it difficult for radical excision [12]. The location and the extent of the lesion usually determine the neurosurgical approach to be taken. Some of the approaches include suboccipital retrosigmoid, retromastoid and subtemporal [12]. Suboccipital retrosigmoid approach is the most preferred as it allows to remove enough tumour and capsule and therefore allow the delay in recurrence of symptoms [12], [13].

Failure to completely remove the cyst will lead to multiple recurrence and cause resurgence of the initial symptoms coupled multiple surgeries. Moreover, incomplete resections have been associated with malignant transformation of EC to squamous cell carcinoma. While this is very rare, it has been described; especially in recurrent EC. The malignant transformation either be suggested by rapid growth or enhancement after contrast administration [12], [16].

Spillage of the cyst contents during surgery may cause chemical meningitis. While steroid is used to treat this, it is usually transient and self-limiting. Furthermore, irrigation of with hydrocortisone solution during the operation and delayed withdrawal of steroids in the post-operative period have been advocated as possible measures for preventing chemical meningitis. This was done in all the cases included in this study. Communicating hydrocephalus can develop as a result of meningitis and might require CSF diversion procedures [12], [15].

## 5 Conclusion

Epidermoid cysts of the brain are rare and still remain a preoperative clinico-radiological conundrum as they may be indistinguishable from other intracranial tumours. “A multidisciplinary approach to these cases is important to improve patient's outcome. One of the challenges in the African continent which may lead to under-reporting of these cases could be access to health care facility, especially neurosurgical services and access to radiological imaging. This study will further assist in improving the awareness of these inracranial cysts by the neurosurgeons and radiologists from the African continent.”

## Patient (parent's) consent

Written informed consent was obtained from the patients and patient's parents for publication of this case report and accompanying images. A copy of the written consent is available for review by the Editor-in-Chief of this journal on request.

## Ethical approval

Ethical Approval was provided by the authors institution.

## Sources of funding

N/A.

## Author contribution

M.C.K. conceptualized the report. M.C.K. and N.A.D and wrote the manuscript. All authors have read and approved the submitted version of this manuscript.

## Guarantor

MC Khaba.

## Research registration

N/A.

## Provenances and peer review

Not commissioned, externally peer reviewed.

## Declaration of competing interest

N/A.
